# Intraoperatively available 3D vascular reconstruction navigation in robotic and laparoscopic right hemicolectomy with complete mesocolic excision: a systematic review (2000–2025)

**DOI:** 10.1007/s10151-026-03301-z

**Published:** 2026-05-09

**Authors:** V. Milovanov, V. Benjamin, Y. M. A. Mohsen, A. A. P. Slesser

**Affiliations:** 1 The Hillingdon Hospitals NHS Foundation Trust, Uxbridge, UK; 2 Imperial College London, London, UK

**Keywords:** Right hemicolectomy, Complete mesocolic excision, Augmented reality, Three-dimensional vascular reconstruction, Robotic colorectal surgery, Surgical navigation

## Abstract

**Background:**

Minimally invasive right hemicolectomy (RH) with complete mesocolic excision (CME) is technically demanding due to variable mesenteric vascular anatomy and the need for precise dissection. Intraoperative visualisation tools such as three-dimensional (3D) reconstructions, augmented reality (AR) and mixed reality (MR) are increasingly used to address these challenges. This review evaluates current evidence on their operative performance, safety and oncologic metrics.

**Method:**

Following Preferred Reporting Items for Systematic Reviews and Meta-Analyse (PRISMA) 2020 guidelines, a PubMed search (updated July 2025) identified English-language studies from 2000 to 2025 reporting intraoperative use of 3D vascular models, AR or MR guidance in robotic or laparoscopic RH. A total of 5 studies comprising 284 RH cases were included: 1 randomised trial, 2 comparative cohorts and 2 feasibility series. Data was extracted on operative time, blood loss, lymph node yield, conversions, complications and user feedback. A narrative synthesis and comparative table summarise the findings.

**Results:**

Across the studies, 1–44 RH cases with 3D guidance were reported (total cohorts: 3–112). With 3D assistance, operative time and blood loss were unchanged or reduced: one matched study reported 3 mL versus 10 mL of blood loss (*p* < 0.001). Lymph node yield was maintained (~28–33 nodes), with one study noting improved central node retrieval. Conversion and complication rates were also unchanged or improved. Surgeons consistently reported enhanced anatomic orientation and procedural confidence.

**Conclusions:**

Intraoperative 3D vascular reconstructions are emerging as feasible and potentially valuable aids in RH with CME. Early evidence suggests improved vascular identification and reduced blood loss without compromising oncologic quality. Further studies are needed to confirm long-term benefits and broader applicability.

## Introduction

Complete mesocolic excision (CME) with central vascular ligation for right-sided colon cancer demands precise identification and division of the mesenteric vasculature. First described by Hohenberger in 2009, CME demonstrated improved oncological outcomes but raised the technical challenge of thorough D3 lymphadenectomy around the superior mesenteric vessels [1]. Intraoperative haemorrhage from high central vessel dissection remains a major risk in CME. Even in experienced hands, variability in mesenteric vascular anatomy can make laparoscopic or robotic CME demanding, especially given the limited tactile feedback and confined view in minimally invasive surgery. Improved visualisation tools are therefore critical to navigate the complex arterial and venous anatomy of the colonic mesentery.

Three-dimensional (3D) vascular mapping has emerged as a strategy to enhance the surgeon’s understanding of patient-specific anatomy before and during surgery. Preoperative contrast-enhanced computed tomography (CT) angiography with 3D reconstructions can generate a “vascular roadmap” of the colon’s blood supply. Early evidence suggested that the availability of this roadmap could reduce errors: Mari et al. demonstrated in 2012 that surgeons who reviewed a 3D CT model of the mesenteric vessels experienced significantly fewer intraoperative vessel identification difficulties and vascular injuries during laparoscopic colectomies [2]. With advances in computer graphics and display technologies, augmented reality (AR) and mixed reality (MR) systems now allow these 3D vascular models to be superimposed or displayed in real-time during surgery [3]. For example, Volonté et al. (2013) reported a pioneering case of robotic right colectomy where a stereoscopic 3D vascular model was integrated into the surgeon’s console view using TilePro and OsiriX software [4]. The tumour and vascular anatomy appeared in 3D within the operative field, improving depth perception and compensating for the lack of haptic feedback in robotics. Such AR navigation tools are hypothesised to shorten the learning curve and enhance safety in CME by clearly delineating critical structures that are otherwise hidden beneath fat and tissue.

In recent years, several pilot studies and trials have evaluated intraoperative use of patient-specific 3D models, AR displays, and holographic guides in right hemicolectomy (RH). These range from small feasibility studies using tablets or headsets to larger comparative studies assessing whether 3D guidance translates into better clinical outcomes [5, 6]. An updated synthesis of this literature is needed to inform surgical practice: earlier reviews have shown AR’s promise in surgical training and other fields, but focussed analyses in colorectal CME are sparse [7–10]. This systematic review, therefore, aims to update the current evidence, up to 2025, on intraoperative 3D vascular reconstruction navigation in robotic and laparoscopic right hemicolectomy. Specifically, we examine whether incorporating 3D vascular models (viewed on monitors, tablets or see-through AR devices) improves operative metrics (time, blood loss, conversions), surgical precision (vessel identification, lymph node harvest) and patient outcomes (complications), as well as how surgeons have subjectively rated these technologies. By adhering to Preferred Reporting Items for Systematic Reviews and Meta-Analyse (PRISMA) 2020 guidelines and focussing on right hemicolectomies with CME, we present a focussed analysis of the practical impact and clinical significance of AR-guided navigation in this complex oncologic procedure.

## Methods

Protocol and inclusion criteria: This review was conducted according to PRISMA 2020 guidelines [11]. The inclusion criteria were defined using the Population, Intervention, Comparison, Outcomes, Study design (PICOS) framework. We included studies involving adult patients undergoing right hemicolectomy with CME for colon neoplasia (Population) where the surgeons had intraoperative access to a 3D reconstruction of the patient’s mesenteric vasculature for navigational assistance (Intervention). This included augmented reality (AR), mixed reality (MR), or other image overlay techniques, as well as cases where a 3D vascular model (e.g. from CT angiography) was available on a screen during surgery. Comparators (Comparison) could include either a control group without 3D guidance (in comparative studies) or none (in single-arm feasibility studies). Outcomes of interest were operative performance measures (operative time, blood loss, conversion to open surgery), oncologic quality measures (lymph node yield) and safety (intra- or postoperative complications). Given the emerging nature of this technology, we included all study designs (Study design) that met these criteria, ranging from randomised controlled trials and cohort studies to case series. We excluded studies without intraoperative use of the 3D model (e.g. if the model was only used for preoperative planning and not referenced during the actual operation) and conference abstracts or non-peer-reviewed reports.

Search strategy: A comprehensive literature search of PubMed was performed, last updated on 9 July 2025. The strategy combined terms for augmented/mixed reality and 3D modelling with terms for right hemicolectomy, using Boolean operators. For example, the PubMed query used was: (augmented reality OR mixed reality OR holographic OR image overlay OR 3D model OR 3D reconstruction) AND (right hemicolectomy OR right colectomy OR complete mesocolic excision) with filters for English language and publication dates 2000/01/01 through 2025/12/31. Reference lists of relevant articles and prior reviews were also cross-searched. After removing duplicates, titles and abstracts were screened for relevance. Full texts of potentially eligible studies were then retrieved and assessed against the inclusion criteria.

**Fig. 1:**
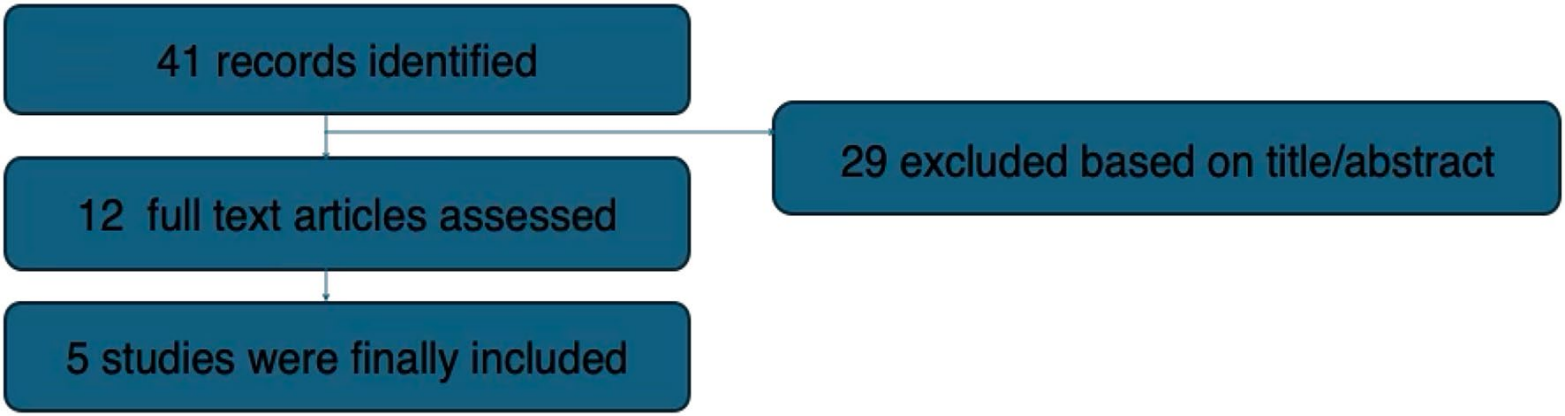
PRISMA flow diagram illustrating the study selection process; a total of five studies met the inclusion criteria for this review

Study selection: The database search yielded 41 records. After deduplication and restriction to English-language full-text peer-reviewed studies, 36 abstracts were screened by a single reviewer. Strict inclusion criteria were applied: (1) adult patients undergoing right hemicolectomy with complete mesocolic excision (CME); (2) intraoperative use of a patient-specific 3D vascular reconstruction (CT angiography-based, AR, MR or comparable technology); (3) laparoscopic or robotic approach; and (4) reporting at least one quantitative operative or oncologic outcome. Exclusion criteria were: (1) studies where 3D models were only used for preoperative planning with no intraoperative reference; (2) colorectal procedures other than right hemicolectomy with CME; (3) reviews, conference abstracts, technical notes without outcome data and non-peer-reviewed studies; and (4) non-English language publications. In total, 12 full texts were assessed; 7 were excluded (4 lacked intraoperative use, 2 were non-CME colectomies, 1 had no extractable outcomes). Five studies met all criteria and were included in the final synthesis. (Fig. 1). These five represent the available peer-reviewed reports (from 2012 to 2025) specifically focussed on intraoperative 3D vascular navigation in right hemicolectomy with CME.

Data extraction and synthesis: Data from each included study were extracted using a standardised form. Extracted variables included study design, number of patients (and whether a control group was present), details of the 3D model technology (source of imaging, software/hardware used, display method), surgical approach (laparoscopy or robotic) and quantitative outcomes (operative times, estimated blood loss or intraoperative bleeding events, conversion rates, lymph node yields and complications). Where studies provided comparative data between 3D-guided and standard surgery, we recorded the reported differences and *p*-values. We also noted any qualitative observations, such as surgeon feedback or learning curve effects. Given the heterogeneity of study designs and outcomes, a meta-analysis was not appropriate. Instead, we performed a narrative synthesis, organising the findings by outcome domain and by study. A comparative summary table (Table 1) was constructed to juxtapose key outcomes across all five studies for clarity.

**Table 1:** Condensed summary table

Study	*N* (3D/control)	Operative time (min) (3D/control)	Blood loss (3D/control)	Lymph node yield (3D/control)	Conversions/complications (3D/control)
Mari et al. 2013 [2]	17/21	130/147 *p* = 0.027	6/19 (haemorrhage events) *p* = 0.006	Not reported	0–1 Complications
Guerriero et al. 2018 [14]	1/2	Not reported	Not reported	Not reported	0–0
Kearns et al. 2024 [12]	22/49	216/157 *p* < 0.01	Not reported	30/28 Mean *p* > 0.05	0–0
Bracale et al. 2024 [15]	3/7	Not reported	Not reported	Not reported	0–0
Ryu et al. 2025 [13]	44/44	289/298 *p* = 0.71	3/10 (ml) *p* < 0.001	33.5/27.5 Median *p* = 0.19	0%/6.8% Conversions

Quality assessment: Due to heterogeneity in study designs and limited numbers, formal risk-of-bias tools (e.g. Cochrane RoB 2, ROBINS-I) were not uniformly applied. However, potential sources of bias were considered systematically, including randomisation (present in Mari et al.), matching (used by Ryu et al.), blinding (often absent), selective reporting and small sample sizes. These elements were appraised qualitatively for each study and discussed in the limitations section.

## Results

### Study characteristics

Five studies published between 2012 and 2025 met the inclusion criteria. The included studies were as follows: one randomised controlled trial by Mari et al. (17 RH cases with 3D CT, *N* = 112) [2]; two comparative cohorts: Kearns et al. (22 RH cases with 3D CT, *N* = 71) [12] and Ryu et al. (44 RH cases with 3D CT, *N* = 88 matched) [13]; and two feasibility series: Guerriero et al. (1 RH case with 3D CT, *N* = 3) [14] and Bracale et al. (3 RH cases with 3D guidance, *N* = 10) [15]. All reported intraoperative use of 3D vascular models during right hemicolectomy with complete mesocolic excision, using monitors, tablets or AR headsets. Three studies used laparoscopic surgery and two incorporated robotics. Model formats included 3D CT angiograms, tablet-based virtual reality (VR) and HoloLens holograms. All procedures targeted central vascular ligation of the ileocolic and colic vessels, with most performed for malignancy (see Table 2 for full study details).

### Operative time

Three studies reported comparative data on operative time. Mari et al. showed a significant reduction from 147 to 130 min with 3D CT guidance (*p* = 0.027) [2], and Romano et al. (not included in final five) reported a reduction from 253 to 225 min using 3D models (*p* ≈ 0.02) [16]. Ryu et al. observed no difference (289 versus 298 min; *p* = 0.71) [13], whilst Kearns et al. reported an increase from 157 to 216 min (*p* < 0.01) early in the learning curve [12]. The two feasibility studies did not report operative duration [14, 15].

### Blood loss and vascular control

Ryu et al. reported a median blood loss of 3 mL with AR versus 10 mL in controls (*p* < 0.001) [13]. Mari et al. recorded intraoperative bleeding in 6 3D-guided versus 19 control cases (*p* = 0.006) [2]. No AR cases required conversion due to bleeding, compared with 3/44 controls (6.8%) in Ryu’s study. Guerriero and Bracale reported no significant bleeding [14, 15], and Kearns noted no measurable difference [12]. Across all studies, there were no reports of increased haemorrhage with AR/3D guidance.

### Lymph node harvest

Mean or median lymph node yield was comparable or slightly higher in the 3D groups: Kearns reported 30 versus 28 nodes, and Ryu 33.5 versus 27.5 (not statistically significant) [12, 13]. Ryu also noted a qualitative increase in central D3 node retrieval with AR. Mari did not report node counts per group, whilst Bracale and Guerriero confirmed oncologic adequacy without numeric data [2, 14, 15]. No study reported a decrease in nodal yield with 3D use.

### Conversion to open surgery

Ryu’s cohort had 0% conversions (0/44) in the AR group versus 6.8% (3/44) in controls [13]. Kearns and Mari reported zero conversions across both groups [2, 12]. Guerriero and Bracale’s series were completed laparoscopically or robotically without conversion [14, 15]. No study suggested AR guidance increased conversion rates; all reported either no difference or a favourable trend.

### Complications and safety

Intraoperative complications occurred in 0 3D versus 3 control cases in Mari’s RCT [2]. Postoperative complication rates were low: Romano reported 2.7% with 3D versus 4.2% without (*p* = 0.04) [16]; Ryu found shorter median hospital stay in AR patients (8 versus 9 days, *p* < 0.01) [13]. No study recorded AR-related adverse events. Guerriero and Bracale confirmed uneventful courses in all cases, and no increase in anastomotic leak or major morbidity was reported [14, 15].

### Surgeon feedback

Surgeons across all studies reported improved anatomical understanding with 3D tools. Ryu’s team noted safer dissection and more confident vascular ligation, with no added mental workload [13]. Kearns and Bracale emphasised educational benefits for trainees [12, 15], whilst Guerriero highlighted a missed vessel detected only on the 3D model [14]. Across all five studies, feedback was uniformly positive regarding utility, ease of use and integration into workflow (Table 2).

**Table 2:** Key outcomes and characteristics of included studies (3D-guided right hemicolectomy with CME)

Study (year)	Patients (study design and approach)	3D model technology used	Operative time	Blood loss	Lymph nodes harvested	Conversions and complications	Surgeon feedback/utility
Mari et al. (2013) [2]	*N* = 112, 17 3D CT RH (RCT: laparoscopic RH, LH, rectal; 3D CT versus no-3D CT) 38 total RH cases: laparoscopic (17 with 3DCT, 21 no-3D CT)	CT angiography with 3D reconstruction (viewed on OR monitor); Standard laparoscopy	RH: 130 versus 147 min (*p* = 0.027) with 3D versus control. LH/rectal also shorter with 3D	Quantitative EBL not given. Fewer haemorrhage events: 6 versus 19 cases bled (*p* = 0.006) with 3D versus control	Not reported (assumed adequate in all cancer resections)	No intraoperative comps with 3D versus 1 comp without; no difference in postoperative comps (0 versus 1 minor). No conversions in either arm	3D roadmap improved surgeon confidence in vessel identification; prior vascular mapping seen as a clear advantage
Guerriero et al. (2018) [14]	*N* = 3, 1 3D CT RH (Feasibility case series; included 1 laparoscopic RH case and 2 other colonic cases)	Patient-specific 3D virtual reality model (CT/MRI-based) on a tablet, manipulated in real-time during laparoscopy	Not reported (feasibility study, no formal timing)	Not reported (minimal blood loss in all cases)	Not reported (oncologic resections deemed adequate; no quantification)	No conversions; no AR-related complications. All surgeries completed uneventfully	3D “virtual clone” model helped identify an anomalous vessel missed on routine imaging, aiding a safer dissection. Demonstrated technical feasibility of intraoperative VR planning
Kearns et al. (2024) [12]	*N* = 71, 22 3D CT RH (Comparative study: 22 laparoscopic RH with 3D versus 49 standard lap RH; all CME with central ligation)	Patient-specific 3D vascular and tumour models from CT, displayed on a nearby screen during surgery (no AR headset)	216 versus 157 min with 3D versus control (*p* < 0.01). Initial cases slower with 3D (learning curve), times improved as team gained experience	Not reported in detail; authors note no significant difference in blood loss between groups (values not given)	30 versus 28 nodes (mean, 3D versus control; no significant difference). Oncologic adequacy maintained with 3D	No conversions in either group. No increase in surgical morbidity with 3D (complication rates similar, no leaks in either arm)	Surgeons and trainees valued the 3D guidance, citing improved anatomy understanding. Some initial workflow disruption (reflected in longer time) but no long-term issues. 3D use became smoother over time, proving practically useful for orientation
Bracale et al. (2024) [15]	*N* = 10, 3 3D CT RH (Narrative report of AR use in colorectal cases; included 3 robotic RH with CME using AR)	Mixed reality hologram via HoloLens headset (preoperative CT-based 3D model projected into operative field). Robotic approach for RH cases	Not reported (focus on feasibility/qualitative outcomes)	Not reported (no significant bleeding events described)	Not reported (no quantitative data; all cases had satisfactory LN harvest per pathology)	No AR-related or unexpected complications. Zero intraoperative adverse events attributed to AR. No conversions (all three RH completed robotically with AR)	AR holograms were easy to integrate and did not increase workload. Helped in tumour localization and assessing invasion, sometimes altering surgical strategy (e.g. confirming tumour extent so as to avoid overly broad resection). Surgeons reported improved 3D comprehension of anatomy
Ryu et al. (2025) [13]	*N* = 88, 44 3D CT RH (Retrospective cohort: 44 laparoscopic RH with MR guidance versus 44 matched laparoscopic RH controls; all D3 CME)	Mixed reality AR using HoloLens 2 + Synapse VINCI/HoloLens software to display holographic 3D vasculature adjacent to surgeon’s console view	289 versus 298 min (AR vs control, *p* = 0.71). No significant difference in operative time. AR did not slow the procedure	3 versus 10 mL EBL (median, AR versus control, *p* < 0.001) – significantly lower with AR. Better visualization likely minimized bleeding	33.5 versus 27.5 nodes (median, AR versus control, *p* = 0.19); statistically similar total yield. More central nodes (middle colic station) retrieved with AR guidance, indicating a more thorough dissection	Conversions: 0% versus 6.8% (AR versus control; trend towards fewer conversions with AR). Postoperative: median hospital stay 8 versus 9 days (*p* < 0.01) favouring AR. Overall complications were low and similar between groups; no AR-related complications reported	Surgeons noted AR made D3 dissection more controlled, with no increase in cognitive load. Concluded that AR “enhances surgical precision” and may have oncologic benefit by ensuring thorough nodal clearance

All studies featured intraoperative availability of a patient-specific 3D vascular model. “3D versus control” indicates comparisons between the group with 3D guidance and the group without (when applicable). *NR* not reported, *AR* augmented reality, *MR* mixed reality, *VR* virtual reality, *CME* complete mesocolic excision, *RH* right hemicolectomy

Key: *RH* right hemicolectomy, *LH* left hemicolectomy, *RCT* randomized controlled trial, *3D CT* three-dimensional computed tomography (angiography), *EBL* estimated blood loss, *AR* augmented reality, *MR* mixed (augmented + virtual) reality

## Discussion

This systematic review provides an up-to-date synthesis of how intraoperative 3D vascular reconstruction navigation is influencing robotic and laparoscopic right hemicolectomy with CME. The findings from five studies spanning a decade of innovation indicate that augmented reality and related 3D visualisation technologies are moving from concept to clinical reality. Here, we discuss the practical implications of these findings, put them in context of colorectal surgical oncology and consider the future directions of this technology.

Surgeons benefit from a more precise understanding of mesenteric anatomy when using intraoperative 3D models, which externalise complex vascular layouts onto a visual display. This enhanced visualisation reduces the cognitive burden of mentally reconstructing vessel orientation from 2D imaging, especially in anatomically variable cases. By clearly depicting key branches such as the ileocolic, right colic and middle colic vessels, these models help avoid misidentification and unnecessary dissection, as demonstrated in studies by Mari and Romano [2, 16]. Moreover, they support safer central vascular ligation by accurately showing the take-off points from the superior mesenteric vessels, minimising the risk of bleeding or vessel avulsion. Ryu et al. further reported improved retrieval of D3 central lymph nodes using AR, indicating that such technology not only improves technical safety, but may also enhance oncologic thoroughness [13].

From a workflow perspective, our review indicates that after an initial adjustment period, AR/3D integration does not slow surgeons down. The one study showing increased time (Kearns) explicitly ties it to early use of technology, akin to the initial learning curve of laparoscopic surgery itself. The fact that Ryu’s large series showed no time penalty implies that surgeons can adapt these tools without sacrificing efficiency [13]. This is a crucial practical point: many surgeons worry that technical novelties in the operating theatre may prolong anaesthesia or distract from the task. The current evidence should alleviate some of those concerns. Moreover, the significant *decrease* in operative time seen in Mari et al. (and in the 3D planning arm of Romano et al.) suggests that in certain scenarios, 3D navigation in fact accelerates the procedure [2, 16]. This was attributed to fewer time-consuming errors or re-routing: for example, avoiding the need to search for a missing artery that turns out to be an aberrant branch. For busy surgical practices, time savings can translate to better utilisation of theatre resources and potentially less anaesthesia exposure for patients.

Robotic platforms offer a unique synergy with AR since the surgeon is already immersed in a console. An AR overlay can be directly integrated into the console view (as Volonté et al. demonstrated in principle) or displayed as a nearby hologram [4]. The robotic setting eliminates the need for additional screens or workflow changes (as the surgeon does not need to look away from instruments to see the AR image), potentially making AR even more seamless. Our included robotic experiences (Ryu and Bracale’s AR cases) indicate that AR is highly compatible with robotics – no interference with robotic instruments was reported. On the contrary, it complements the precision that robotics provides [13, 15]. One practical implication is that AR might help compensate for the lack of haptic feedback in robotics. Since the surgeon cannot feel tension on tissues in robotic surgery, AR provides a visual cue of vascular structures and can prevent inadvertent tugging on a short vessel that one might have sensed by feel in open surgery. AR essentially enriches the sensory input for robotic surgeons (vision replacing some of the lost touch). This enhanced visual–spatial awareness could partially explain why Ryu’s laparoscopic AR group experienced such minimal blood loss.

Positive trainee feedback and improvement in knowledge scores with 3D models highlight an often under-appreciated benefit: education. In complex oncologic surgery such as CME, understanding the vascular anatomy is a steep learning curve for residents and fellows. The use of 3D models in training sessions [17] and even during the operation can accelerate learning. Trainees can correlate the live surgical field with the 3D map, reinforcing their spatial understanding. This may ultimately improve surgical training outcomes and produce surgeons who are more adept at CME. From a clinical perspective, a well-trained assistant or secondary surgeon who understands the anatomy can better assist during the case (e.g. in laparoscopic cases, the camera can be guided or retracted with more insight). Thus, the integration of these technologies could raise the overall performance of the surgical team, not just the primary surgeon.

Whilst this review focusses on intraoperative and short-term outcomes, the potential impact on longer-term patient outcomes is noteworthy. Augmented reality (AR) may contribute to reduced complications by minimising intraoperative blood loss and vascular injuries, potentially lowering transfusion needs, conversion rates and promoting faster recovery, reflected in shorter hospital stays reported in some studies. Although none of the included studies were powered to detect rare complications such as anastomotic leak, importantly, none reported an increase in adverse events with AR use. From an oncologic standpoint, complete mesocolic excision (CME) relies on meticulous central dissection, and AR appears to support this rigour by enhancing the clarity and confidence with which surgeons approach vascular anatomy. By enabling precise ligation and thorough node clearance, especially in D3 stations, AR may hypothetically improve long-term outcomes such as recurrence or survival. However, no current evidence demonstrates such long-term benefits – these remain speculative until confirmed by future studies. Additionally, AR facilitates a move towards individualised, anatomy-specific dissection – allowing surgeons to tailor resections on the basis of unique vascular patterns as opposed to generic templates. This precision may reduce unnecessary tissue trauma and ensure oncologic completeness, exemplifying the principles of patient-specific surgery.

This review adhered to PRISMA 2020 and used a structured PICOS framework, but some limitations must be acknowledged. Screening and data extraction were performed by a single reviewer, introducing potential selection bias. The studies included were heterogeneous – differing in AR modality (static versus real-time, monitor versus headset), surgical technique (laparoscopic versus robotic) and study design (RCT, cohort, feasibility). Most sample sizes were small (typically fewer than 25 AR cases), limiting generalisability and statistical power. These factors should be considered when interpreting the strength of the conclusions.

The encouraging results of this review highlight several directions for future development. Larger, prospective studies – ideally multi-centre randomised controlled trials – are needed to confirm whether the benefits of AR-guided CME hold across broader populations and to detect rare complications. Long-term follow-up data, including recurrence and survival rates, will be essential to assess oncologic equivalence or superiority. Technological enhancements, such as real-time model updates using intraoperative imaging and improved image registration, could make AR more adaptive and responsive during surgery. Artificial intelligence may play a future role by automating vessel recognition or alerting surgeons to unseen structures. Finally, ongoing user experience research will be critical to refining AR interfaces to become as intuitive and seamless as conventional surgical views.

Despite promising findings, several limitations affect the interpretation of this review. The number of studies is small, with limited sample sizes – most involving fewer than 25 AR-guided cases – reducing power to detect rare complications or generalise outcomes. Study heterogeneity also limits comparability, with mixed designs (RCTs, cohort studies, case series), varied platforms (laparoscopic versus robotic) and different AR technologies (monitors, tablets, headsets). Selection and publication bias may exist, as studies with positive outcomes are more likely to be published, and retrospective designs may skew patient inclusion. Learning curve effects were evident – early adopters such as Kearns reported longer operative times that improved with experience [12]. Importantly, long-term oncologic outcomes remain unknown, as current data focus only on intraoperative and short-term endpoints. Many studies lacked detailed reporting on blood loss, node counts or complication rates, leaving data gaps. Furthermore, most studies were conducted in high-resource centres with advanced technical support, limiting generalisability to smaller settings. The technology itself is rapidly evolving, thus findings may soon be outdated as newer systems improve usability and accuracy. These caveats underscore that whilst AR-guided CME appears feasible and safe, broader validation is needed before widespread adoption.

## Conclusions

Augmented reality (AR) and 3D vascular reconstruction are emerging as potentially valuable tools in robotic and laparoscopic right hemicolectomy with complete mesocolic excision (CME). This review of studies from 2000 to 2025 demonstrates their feasibility and safety, with no AR-related complications and smooth integration into surgical workflows. Operative outcomes such as blood loss, vascular injury and conversion rates were improved or remained unchanged, and oncologic standards were preserved – lymph node yields were consistent, with a possible improvement in central nodal dissection. Surgeons consistently reported enhanced anatomical clarity and confidence, particularly during vascular ligation, indicating that AR may support improved performance and surgical training. Clinically, AR-guided CME has the potential to reduce intraoperative risk and variability, enabling more precise, patient-specific surgery. Clinically, AR-guided CME shows promise in reducing intraoperative risk and variability. However, due to the very limited data, its effect yet remains speculative and requires further validation. Further research is necessary to validate long-term oncologic outcomes, define learning curves and assess cost-effectiveness before widespread adoption can be recommended.

## Data Availability

No datasets were generated or analysed during the current study.
